# Challenges in Developing a Patient-Reported Symptom-Based Risk Stratification System for Suspected Head and Neck Cancer: Protocol for a Qualitative Case Study

**DOI:** 10.2196/74262

**Published:** 2025-11-25

**Authors:** Chinasa Odo, Joanne Patterson, Nikki Rousseau, Vinidh Paleri, Rebecca Randell

**Affiliations:** 1 Centre for Digital Innovations in Health & Social Care, University of Bradford, Richmond Rd, Bradford, BD7 1DP, United Kingdom, +44 01274232323; 2 Wolfson Centre for Applied Health Research, Bradford, United Kingdom; 3 School of Health Sciences, University of Liverpool, Liverpool, United Kingdom; 4 Leeds Institute of Clinical Trials Research, University of Leeds, Leeds, United Kingdom; 5 The Royal Marsden NHS Foundation Trust, London, United Kingdom

**Keywords:** challenges, head and neck cancer, patient-reported symptoms, risk stratification, symptom-based

## Abstract

**Background:**

The Symptom Input Clinical (SYNC) system is being developed to enhance the timely reporting of head and neck cancer symptoms and ensure that high-risk patients receive faster diagnoses. A key feature of the system is a digital questionnaire co-designed with patient representatives to accommodate varying levels of digital literacy. The system integrates a validated algorithm that assigns risk scores to categorize cases as low or high risk and a dashboard that supports clinicians by providing them with patient reports. However, the development process has encountered challenges that necessitate a systematic evaluation of the process, roles, and experiences of team members.

**Objective:**

This study aims to identify challenges faced during development, how these challenges were addressed, and the implications for future digital health innovations.

**Methods:**

A qualitative single–case study approach will be used following the Standards for Reporting Qualitative Research guidelines focusing on individuals involved in the SYNC system’s development. Participants will be selected using a combination of purposive and snowball sampling to ensure diverse perspectives, using meeting minutes and recommendations from key stakeholders. A total of 8 to 12 participants will be interviewed, representing clinical, research, and IT roles. Data collection will involve semistructured interviews, which will be conducted through Microsoft Teams. The interviews are expected to last between 40 and 60 minutes each. These interviews will be audio recorded, transcribed, and analyzed using framework analysis in Dedoose. The actor-network theory will guide the analysis by mapping interactions between human and nonhuman actors, such as developers, clinicians, and technological tools, to understand how they influenced the project’s outcomes. Participant confidentiality will be maintained through data encryption, deidentification, and secure storage.

**Results:**

We anticipate identifying key barriers and facilitators in the SYNC system’s development, including technical, organizational, and collaboration-related challenges. The findings are expected to provide a detailed account of the challenges encountered, such as delays, security concerns, and coordination issues; an insight into how these challenges were mitigated; and lessons learned and recommendations for improving digital health technology development, including best practices for co-design, technical integration, and stakeholder engagement.

**Conclusions:**

This case study will provide valuable insights into the complexities of developing digital health technologies, particularly in collaborative, multistakeholder environments. Documenting the challenges encountered in the SYNC system’s development will contribute to best practices in digital health innovation.

## Introduction

The Symptom Input Clinical (SYNC) system was developed to enhance the timely reporting of head and neck cancer (HNC) symptoms and ensure that high-risk patients receive faster diagnoses. It was developed in response to delays in the United Kingdom’s “two-week wait” cancer referral pathway, which mandates that patients suspected of cancer be seen by a specialist within 2 weeks of general practitioner referral [1]. This process of referral does not stratify patients by actual risk level, meaning that high-risk individuals may wait alongside those with low clinical urgency, potentially delaying diagnosis and treatment [2].

The SYNC system is a clinician-led initiative designed in collaboration with patients and other stakeholders to improve risk-based triage. A Microsoft Excel–based version innovation, with clinicians entering information based on patient responses to obtain a risk score, was piloted in National Health Service settings across secondary care. The SYNC system is intended to improve upon this. Patients complete a digital symptom questionnaire upon referral; their responses are assessed via a validated algorithm to classify cases as low or high risk [3]. The system enables clinicians to access symptom and risk information prior to consultation, supporting earlier intervention for high-risk cases. The SYNC system includes a dashboard designed to support clinicians and cancer care service management teams by enabling them to monitor where the patient is in the pathway, including patients who have received, completed, and returned the questionnaire.

To optimize the design of the digital questionnaire, it was co-designed with relevant stakeholders, specifically patient representatives, to capture symptoms reported by patients in an accessible format to accommodate a range of health and digital literacy levels [3-8]. Patient representatives were recruited to participate in co-design and usability testing activities as part of the development of the SYNC digital questionnaire. Some of these individuals had prior experience with the urgent suspected cancer referral pathway for HNC and were engaged to provide lived experience perspectives. As co-design collaborators, patient representatives contributed to workshop discussions, validated early prototypes, and participated in think-aloud and task-based usability evaluations. Their insights were critical in shaping the content, structure, and interface of the tool, ensuring that it was understandable, user-friendly, and appropriate for patients at various levels of health and digital literacy.

The process of developing the SYNC system encountered numerous delays and challenges. These challenges emphasized the need to systematically study and document the processes, roles, and perspectives of team members to inform future developments in digital health technology. The first step in tackling the challenges is to identify them. This study will capture the key experiences of those involved in the process and planning during the development of the SYNC system. The information gathered in this study will help those planning similar projects in the future.

The aim of this study is to examine the development challenges encountered during the design of the SYNC system and how these influenced the project’s scope, timeline, and implementation dynamics. The objectives are to (1) explore the experiences and roles of key stakeholders, including clinicians, researchers, and SYNC system developers, throughout the design process; (2) identify and analyze the strategies used to address technical, organizational, and communication-related barriers that emerged during development; (3) grounded in actor-network theory (ANT), assess how interactions between human and nonhuman actors (eg, individuals, algorithms, technologies, and institutional workflows) shaped the trajectory and evolution of the SYNC system; and (4) generate transferable insights to inform the design and implementation of future digital health technologies in comparable clinical and organizational settings. These objectives are exploratory in nature and grounded in the hypothesis that inclusive, context-sensitive design processes mediated by sociotechnical interactions play a critical role in shaping the design, usability, and long-term sustainability of digital health care innovations.

## Methods

### Overview

This research will adopt a qualitative single–case study design and adhere to the Standards for Reporting Qualitative Research guidelines by O’Brien et al [9] to ensure transparency and methodological rigor in reporting. Given the case study design and the aim to explore development challenges from a system-wide perspective, the Standards for Reporting Qualitative Research provide the most appropriate framework for guiding the design, conduct, and reporting of this study. A case study is an empirical inquiry that involves the study of an individual unit, such as a person, community, or project, with a focus on developmental factors in relation to their environment [10,11] and involving the collection of multiple forms of data [12]. This study will focus on individuals involved in the development of the SYNC system and will examine cross-team experiences and challenges, exploring how these factors influence the development processes. This method was chosen to enable an in-depth analysis of real-world challenges and the complex interactions among individuals, teams, and the technology.

### Recruitment and Sampling Strategy

Participants will be selected using a combination of purposive and snowball sampling, which is particularly effective for engaging individuals with specific insights into a project’s processes and challenges [13]. This method leverages existing social networks and relationships, enabling us to identify and recruit participants who are knowledgeable about the SYNC system conception and development. We will review minutes of previous meetings, and if someone is repeatedly mentioned or appears to have had a key influence on an element of study progress, we will highlight that individual as key in participant selection. Initial recruitment will involve purposive selection of key individuals involved in the SYNC system’s development, ensuring representation across the various teams involved in the development process (and including individuals with clinical, research, and IT roles). They will be asked to recommend colleagues they believe could provide valuable input. In this way, stakeholders with smaller roles in the project will also be considered to ensure a broader range of perspectives and enhance the credibility of the findings. We plan to recruit between 8 and 12 participants from different roles, especially those whose roles in the project were delayed due to challenges encountered. There is no consensus on the number of interviews required to provide an adequate understanding of experiences within a particular setting. However, this range allows for sufficient depth in identifying themes while keeping data collection manageable. This sample size is considered sufficient to reach thematic saturation when exploring experiential dimensions within well-defined stakeholder groups, such as clinicians, researchers, and developers engaged in a co-design project [14]. Saturation will be assessed iteratively during data collection and analysis using the constant comparative method. We will evaluate for saturation when no new codes or themes emerge across at least 2 consecutive interviews or focus groups. A saturation grid will be maintained to document the emergence of themes and monitor redundancy in incoming data. If thematic saturation is not reached within the initial sample, additional participants will be recruited until saturation is achieved.

In case of challenges in recruitment due to potential participants’ work commitments or other concerns, we will extend the recruitment period and be flexible in scheduling interviews (eg, offering them outside of normal working hours).

### Data Collection

This study will use multiple data collection methods that will involve both qualitative interviews and document analysis. Data will be collected through semistructured interviews conducted via Microsoft Teams. This will allow participants the flexibility to take part from their preferred location and avoid the logistical challenges of travel. Interviews will last between 40 and 60 minutes and will be audio recorded with participants’ consent. This approach will ensure a consistent format while enabling researchers to capture rich, detailed narratives. The interviews will focus on participants’ roles, challenges experienced during the SYNC system development, and recommendations for improving similar projects (Multimedia Appendix 1). In addition to conducting interviews, the researchers will undertake review of relevant documents such as meeting minutes and other pertinent records to gather insights into any documented challenges. Participants will also be asked to provide any available documents that could help uncover developmental challenges. This will offer a more comprehensive understanding of the issues.

### Data Analysis

Framework analysis, a qualitative method that uses a structured matrix to organize and analyze data (interview transcripts and collected documents) by case and code [15], will be used in this study. This method will ensure a clear audit trail for credibility and support input from the wider project team, including those with limited qualitative analysis experience. Framework analysis will be conducted using Dedoose (SocioCultural Research Consultants), with codes derived inductively and deductively from ANT [16]. The aim is to understand how people, ideas, technologies, and nature form networks. To ensure dependability, a second researcher will review a sample of the interview transcripts and documents to identify any additional codes. The first researcher will then use the agreed-upon codes to index the data. Coded data will be summarized in a matrix, enabling collaborative discussion regarding patterns in the data to further strengthen the reliability of the findings. Following these consensus meetings, the first researcher will use the matrix to produce narrative summaries of each theme, returning to the original data where necessary. The documentary analysis will be systematically integrated with interview data to cross-validate emerging themes and interpretations.

Guidance for qualitative studies in health informatics will be followed, ensuring that the findings are robust and credible [17]. This guidance adheres to established criteria for transparency and rigor in qualitative research.

### Ethical Considerations

The study protocol for the co-design of the SYNC system was reviewed and approved by the Committee for Clinical Research at the Royal Marsden (CCR5686) and the UK Health Research Authority (Integrated Research Application System 315419). An amendment will be submitted to undertake this additional research. All participants will receive an information sheet and consent form via email outlining the study’s objectives, procedures, and ethical considerations. Participants will be given 48 hours where possible to review the documents and confirm their willingness to take part by responding to the researcher.

To safeguard participant confidentiality, this study will implement encryption for interview recordings during transmission and storage. In publications, the researcher will avoid reporting specific details that could single out a participant. Interview recordings will initially be stored on the University of Bradford secure servers and transcribed using Microsoft Teams. Following an interview, the transcript will be checked against the recording for accuracy, and once complete, the recording will be deleted. Interview transcripts will be uploaded to Dedoose, a secure and collaborative research and evaluation data analysis application, and access will be restricted to the researchers involved through role-based access control and multifactor authentication. All data will be deidentified before analysis to protect participant identities. Data will be stored securely and retained only until the completion of the Evolution of a Patient-Reported Symptom-Based Risk Stratification System to Redesign the Suspected Head and Neck Cancer Referral Pathway (EVEREST-HN) study, with a clear policy for deletion based on the University of Bradford storage and retention policy. The informed consent form will outline how data will be handled, and participants will be informed of their rights regarding data withdrawal.

### Theoretical Framework: ANT

The design and conduct of this study will be guided by ANT [16]. ANT challenged the traditional dichotomies, dissolving artificial boundaries among technology, organization, nature, and society, focusing on the interconnected networks of human and nonhuman actors that shape and are shaped by each other. ANT provides a framework for understanding how both human (eg, developers, clinicians, and patients) and nonhuman (eg, the SYNC system, organizational tools, and processes) actors interact within a network to influence the outcome of the project development. In the context of the SYNC system, ANT will help uncover how these interactions shaped the project, such as how collaboration between diverse teams impacted decision-making and how technical challenges influenced workflow. By mapping these relationships, ANT will highlight critical factors contributing to the system’s successes and challenges, offering insights for improving future digital health projects. These ANT concepts have informed the interview questions and will inform the codes used to index the transcripts and project documents.

ANT will be particularly useful in identifying how roles and responsibilities were distributed among team members and how these roles evolved over time. It will also help uncover the influence of technical and organizational factors on the project’s trajectory, offering valuable insights for refining processes in future digital health technology development.

## Results

### Overview

This study is expected to provide a comprehensive analysis of the challenges encountered during the development of the SYNC system, as well as the lessons derived from addressing these challenges. Data collection is scheduled to commence in August 2025. At the time of this manuscript submission, participant recruitment has not yet started. The data analysis and the anticipated findings will be formally reported in the first quarter of 2026.

### Potential Limitations of the Proposed Methods

A limitation of the study design is the use of purposive and snowball sampling, which may present several challenges. Purposive and snowball sampling were selected to ensure inclusion of information-rich participants with direct involvement in the system’s development and implementation, aligning with the study’s aim to explore design- and process-level dynamics. There is no comprehensive list of individuals involved in the system’s development, and so snowball sampling is essential to identify other participants. However, reliance on participants to recommend others could introduce selection bias, potentially limiting the diversity of perspectives captured and affecting the generalizability of the findings as insights might reflect only a subset of those involved in the project. For instance, participants may predominantly refer colleagues within their immediate teams or with similar views, which could overlook critical voices or unique experiences. Snowball sampling might not adequately capture perspectives from less connected stakeholders or peripheral team members, thus limiting understanding of complex interactions between technical and organizational factors. The use of minutes as a secondary route to sampling might help mitigate this risk. If critical or dissenting perspectives are underrepresented, this limitation will be noted and reflected in the interpretation of the findings.

Another challenge in small team projects is maintaining participant anonymity, especially when using single-case designs. It may be hard to disguise identities, at least from other people within the case, if not externally. Consequently, participants might worry about being identifiable, which could impact their willingness to share honest feedback. To minimize this, findings will be reported in a more generalized form by combining responses where possible. Additionally, the researchers acknowledge that their backgrounds and experiences may influence the research. Researchers’ positionalities and potential biases will be actively considered throughout the study to enhance transparency and reflexivity. The lead author (CO) brings experience as a qualitative researcher and holds affiliation with the broader EVEREST-HN program, although they were not directly involved in the technical development of the SYNC system. This positioning offers both contextual familiarity and a degree of critical distance. Other members of the research team include system developers, clinical collaborators, and methodological advisors whose varying levels of involvement in the project may influence their interpretations. To mitigate potential biases, reflexive journals [18] will be maintained to document assumptions, role-related insights, and decision-making processes throughout the research. Coding will be conducted collaboratively, with at least one researcher not directly involved in the development of the SYNC system to provide an external perspective. Peer debriefing sessions will be held to challenge assumptions and ensure a balanced interpretation of the findings. Memo writing during analysis will also be used to capture reflections on how individual experiences may shape emerging themes. These strategies will support the credibility and rigor of the analysis while ensuring that findings are grounded in the participants’ perspectives rather than the researchers’ preconceptions.

## Discussion

This protocol describes the methods that will be used for capturing the experiences and challenges of developing the SYNC system, which will offer actionable insights for designing and implementing similar technologies in health informatics. On the basis of a qualitative case study approach, purposive and snowball sampling, and ANT, the findings will address collaboration challenges, promote inclusive co-design, and optimize technical integration. This study is expected to generate important insights into the development of the SYNC system, a digital health innovation designed to support timely symptom reporting and risk stratification for patients referred with suspected HNC. The findings are anticipated to provide a detailed account of the challenges encountered during system development, including technical constraints, interprofessional coordination difficulties, governance processes, and changes in scope or delivery timelines. This study also aims to identify facilitators and adaptive strategies used by the development team to overcome these challenges. These findings will inform future efforts to design, implement, and scale similar digital health systems, particularly in the context of multistakeholder pathways.

While prior research [4,5,19], including the protocol by Albutt et al [3], has focused on the co-design and usability testing of the SYNC patient-facing questionnaire, limited attention has been paid to the underlying developmental processes and system-level barriers. This study addresses that gap by using a qualitative case study approach guided by ANT to examine how human and nonhuman actors, including clinicians, developers, and digital platforms, interacted during system development. In contrast to studies that primarily explore patient engagement or tool efficacy, this work contributes to the growing but underdeveloped literature on digital health implementation processes, organizational complexity, and collaborative design in real-world health care settings. A strength of this study will be its use of multiple data sources and perspectives to capture the layered complexity of system development. The application of ANT as a theoretical framework supports a more nuanced understanding of how actors influence and are influenced by their sociotechnical networks. By engaging participants from clinical, IT, and research backgrounds, this study will provide a richer, more holistic view of development challenges. The use of reflexive practices and team-based analysis will further enhance the trustworthiness of the findings.

This study will provide practical recommendations for future digital health innovations, particularly those implemented in time-sensitive pathways such as cancer referral. The findings may be used to guide best practices in co-design, risk algorithm integration, technical development, and stakeholder engagement. Future research should consider evaluating the impact of the SYNC system and similar digital triage tools after implementation, including effects on diagnostic timelines, patient outcomes, and system-level efficiency. Comparative studies across different disease areas or health care systems could further refine understanding of transferable practices and context-specific adaptations.

To maximize the reach and utility of the study findings, we will pursue a comprehensive dissemination strategy. This includes publication in peer-reviewed journals, presentations at relevant academic and professional conferences (eg, the American Medical Informatics Association, MedInfo, and Health Services Research UK), and targeted knowledge exchange workshops with stakeholders. In addition, we will develop a practical implementation resource summarizing key lessons learned, which will be shared with health care providers, innovation hubs, and digital health funders to inform future technology development and deployment efforts.

## Supplementary material

10.2196/74262 Multimedia Appendix 1 Topic guide: case study interviews.

## Abbreviations

ANT: actor-network theory
EVEREST-HN: Evolution of a Patient-Reported Symptom-Based Risk Stratification System to Redesign the Suspected Head and Neck Cancer Referral Pathway
HNC: head and neck cancer
SYNC: Symptom Input Clinical
