# Correction: Challenges in Developing a Patient-Reported Symptom-Based Risk Stratification System for Suspected Head and Neck Cancer: Protocol for a Qualitative Case Study

**DOI:** 10.2196/100038

**Published:** 2026-06-26

**Authors:** Chinasa Odo, Joanne Patterson, Nikki Rousseau, Vinidh Paleri, Rebecca Randell

**Affiliations:** 1 Centre for Digital Innovations in Health & Social Care, University of Bradford, Richmond Rd, Bradford, BD7 1DP, United Kingdom, 44 01274 232323; 2 Wolfson Centre for Applied Health Research, Bradford, United Kingdom; 3 School of Health Sciences, University of Liverpool, Liverpool, United Kingdom; 4 Leeds Institute of Clinical Trials Research, University of Leeds, Leeds, United Kingdom; 5 The Royal Marsden NHS Foundation Trust, London, United Kingdom

In “Challenges in Developing a Patient-Reported Symptom-Based Risk Stratification System for Suspected Head and Neck Cancer: Protocol for a Qualitative Case Study” [1], the authors noted one error.

Multimedia Appendix 1 has been replaced with the file attached to this notice.

The correction will appear in the online version of the paper on the JMIR Publications website, together with the publication of this correction notice. Because this was made after submission to PubMed, PubMed Central, and other full-text repositories, the corrected article has also been resubmitted to those repositories.

## Supplementary material

10.2196/100038 Multimedia Appendix 1 Topic guide: case study interviews.
