# Supplementary material for: Challenges in Developing a Patient-Reported Symptom-Based Risk Stratification System for Suspected Head and Neck Cancer: Protocol for a Qualitative Case Study
Source: JMIR Res Protoc. 2025 Nov 25;14:e74262. doi: 10.2196/74262 (PMC12646551; doi:10.2196/74262)
Supplement: Multimedia Appendix 1 [file resprot-v14-e74262-s001.docx]

Appendix A: **Topic guide: Case study interviews**

The interview schedule is developmental. The questions will be tailored to each interviewee and additional questions may be added as the research progresses.

**1. Introduction**

- Can you tell me about yourself and describe your role in the SYNC system development project?
- At what stage(s) of the development process did you get involved?

**2. Initial development and transition from M-team to NHS team**

- Why was the development of the SYNC system initially assigned to M-team?
- Can you share insights into the reasons behind transitioning the development to NHS team?
- What challenges arose during this transition (e.g., communication, continuity, technical constraints)?
- How were these challenges addressed, if at all?

**3. Design and development decisions**

- What factors influenced the decision to focus on voice chat and web-based questionnaire, rather than incorporating SMS and WhatsApp?
- Were there any technical or user feedback-related constraints that led to this change?
- Do you think that these changes would impact the user acceptance?

**4. Security and compliance concerns**

- Were there specific challenges regarding data security, information governance (IG) compliance, or user trust?
- How were these concerns addressed during development?

**5. Team dynamics and organisational factors**

- Were there any difficulties in coordinating the teams e.g., developers, user researchers and other stakeholders?
- Was there any conflicting priorities or resource limitations that influenced the development process?

**6. Reflections and lessons learned**

- Looking back, what do you think were the most significant challenges in the development of the SYNC system?
- What would you do differently if you were to embark on a similar project again?
- Are there any key lessons learned from this experience that you believe should be shared with others developing digital healthcare technologies?
